# Potential of two delivery systems for nisin topical application to dental plaque biofilms in dogs

**DOI:** 10.1186/s12917-018-1692-9

**Published:** 2018-11-29

**Authors:** Eva Cunha, Tiago Trovão, Ana Pinheiro, Telmo Nunes, Raquel Santos, Jorge Moreira da Silva, Berta São Braz, Luís Tavares, Ana Salomé Veiga, Manuela Oliveira

**Affiliations:** 1 CIISA - Centro de Investigação Interdisciplinar em Sanidade Animal, Faculdade de Medicina Veterinária, Universidade de Lisboa, Avenida da Universidade Técnica, 1300-477 Lisboa, Portugal; 2 Instituto de Medicina Molecular, Faculdade de Medicina, Universidade de Lisboa, Avenida Professor Egas Moniz, 1649-028 Lisboa, Portugal; 3 Virbac de Portugal Laboratórios, Lda, Rua do Centro Empresarial, Quinta da Beloura, 2710-693 Sintra, Portugal

**Keywords:** Dogs, Enterococci, Guar-gum gel, Nisin, Periodontal disease, Toothpaste

## Abstract

**Background:**

Periodontal disease (PD) is caused by the development of a microbial biofilm (dental plaque) in the periodontium, affecting approximately 80% of dogs. Several bacterial species present in the canine oral cavity can be implicated in the development of this disease, including *Enterococcus* spp. To decrease antibiotic administration, a possible control strategy for dog’s enterococcal PD may involve the use of the antimicrobial peptide (AMP) nisin.

Nisin’s inhibitory activity was evaluated against a collection of previously characterized enterococci obtained from the oral cavity of dogs with PD (*n* = 20), as well as the potential of a guar-gum gel and a veterinary toothpaste as topical delivery systems for this AMP. The Minimum Inhibitory (MIC) and Bactericidal Concentrations (MBC) and the Minimum Biofilm Eradication (MBEC) and Inhibitory Concentrations (MBIC) were determined for nisin and for the supplemented guar-gum gel. For the supplemented veterinary toothpaste an agar-well diffusion assay was used to evaluate its inhibitory potential.

**Results:**

Nisin was effective against all isolates. Independently of being or not incorporated in the guar-gum gel, its inhibitory activity on biofilms was higher, with MBIC (12.46 ± 5.16 and 13.60 ± 4.31 μg/mL, respectively) and MBEC values (21.87 ± 11.33 and 42.34 ± 16.61 μg/mL) being lower than MIC (24.61 ± 4.64 and 14.90 ± 4.10 μg/mL) and MBC (63.09 ± 13.22 and 66.63 ± 19.55 μg/mL) values. The supplemented toothpaste was also effective, showing inhibitory activity against 95% of the isolates.

**Conclusions:**

The inhibitory ability of nisin when incorporated in the two delivery systems was maintained or increased, demonstrating the potential of these supplemented vehicles to be applied to PD control in dogs.

## Background

Periodontal disease (PD) is one of the most frequent and widespread diseases in dogs [1]. It is an inflammatory disease caused by the formation of a microbial biofilm, also known as dental plaque, that affects the periodontium [2]. Its prevalence ranges from 44 to 64%, rising to 85% in animals over 4 years of age [1, 3]. PD is a progressive disease that begins with gingivitis and, if untreated, can develop to periodontitis. Gingivitis, which may be prevented through dental hygiene care procedures and regular professional periodontal treatments [1, 2, 4, 5], begins when bacteria from the oral cavity adhere to teeth surface forming a microbial biofilm, also known as dental plaque [3, 4]. These microorganisms secrete toxins and other metabolic products that invade periodontal tissues, leading to an inflammatory response from the animal’s immune system [2, 4]. This persistent host inflammatory response against the bacterial aggression causes most damage to the periodontium tissue. As inflammation increases and the destruction of the periodontal ligament and alveolar bone occurs, gingivitis can progress to periodontitis, resulting in irreversible histopathological changes such as gingival recession, periodontal pocket formation and eventually tooth loss [1, 4, 6]. In this phase bacteria can spread via bloodstream causing systemic diseases [1, 5, 7, 8].

Dogs’ PD microbiota is complex, and changes in the microbial biofilm can be observed according to PD severity. *Enterococcus* spp. is frequently present in the canine oral cavity, being related with PD development [8, 9]. The prevention of dental plaque formation and its removal are essential steps for PD control and can be achieved by a combination of oral hygiene care procedures, special diet and regular professional periodontal treatments [4, 5, 10]. After PD establishment, initial treatment includes dental plaque removal; surgical measures, aiming at periodontium regeneration, are applied in severe cases [4, 5, 10, 11]. These treatments are usually complemented with antimicrobial therapy, which may contribute for the dissemination of antimicrobial resistance strains [5]. Thus, there is an urgent need to develop novel antimicrobial protocols with a potential application to PD control.

Antimicrobial peptides (AMP) are a promising alternative to conventional antibiotics. These molecules are produced by most organisms as part of their innate immune response against a broad range of pathogens and have been described for their ability to prevent biofilm formation, act on pre-formed biofilms and as modulators of the immune system [12–17]. Nisin, a molecule produced by *Lactococcus lactis,* is an AMP with unusual amino acid residues, such as lanthionine or methyllanthionine, essential for its activity [13, 18, 19]. It is active against Gram-positive bacteria, including multidrug-resistant strains [13, 20, 21], acting by binding to Lipid II and interfering with cell wall biosynthesis, leading to bacterial death [13, 19]. Currently approved by EFSA, WHO, FAO and FDA, and used since 1953 as an additive by the food industry, nisin has the potential to be applied in biomedical research [19]. In fact, several reports describe the potential of nisin application to PD control [18, 19, 22, 23].

Despite all their advantages, AMP successful delivery is a challenge, since they can be inactivated before reaching their target at therapeutic concentrations [24]. Several reports describe natural polysaccharides as promising drug delivery systems due to their non-toxicity, biodegradability and biocompatibility [25, 26]. Guar gum is a natural polysaccharide obtained from *Cyamopsis tetragonolobus*, consisting of a linear polymer of D-galactose and D-mannose [26]. Its properties as thickener, emulsifier, gelling and binder compound, quick solubility in cold water, wide pH stability and film forming ability, make guar-gum an interesting system for bioactive agent’s delivery [25, 26]. Toothpastes are also potential delivery system for AMP topical administration to dogs’ oral cavity. Tooth brushing is an effective mechanical technique to reduce dental plaque [5, 27], and nisin supplementation may improve the efficacy of regular toothpastes for PD prophylaxis [27].

This study aimed to develop and evaluate a novel antimicrobial approach for the control of PD in dogs. For this purpose, the inhibitory activity of nisin against selected enterococci isolated from dogs with PD was determined. Additionally, the potential of a veterinary toothpaste and of a guar-gum gel, as topical nisin-delivery systems, was evaluated.

## Results

### Minimum inhibitory and bactericidal concentration of nisin and of the supplemented guar-gum gel

All the 20 clinical enterococci were considered susceptible to nisin. MIC values for the nisin solution ranged from 8.5 to 26.75 μg/mL, with an average value of 14.90 ± 4.10 μg/mL. When incorporated in the guar-gum gel, nisin MIC values were significantly different (*p*-value < 0.05) and ranged from 17.25 to 33.25 μg/mL. The average value was 24.61 ± 4.64 μg/mL (Tables 1 and 2).

**Table 1:** MIC, MBC, MBIC and MBEC determinations for nisin and supplemented guar-gum gel solutions, against 20 enterococci isolated from dogs with PD

Strain identification	Nisin solution (μg/mL)				Supplemented guar-gum gel solution (μg/mL)			
	MIC	MBC	MBIC	MBEC	MIC	MBC	MBIC	MBEC
M2b								
*E. faecalis*	12.75	73.00	8.25	13.00	21.25	53.75	14.50	29.25
M2c								
*E. faecalis*	15.75	85.50	8.00	31.00	22.50	75	20.25	43.75
M3b								
*E. faecalis*	14.75	60.25	14.00	57.00	17.25	61.00	11.50	22.00
M3d								
*E. faecalis*	15.75	82.25	14.00	50.00	22.00	79.75	15.50	33.25
M4a								
*E. faecalis*	21.50	98.50	15.75	25.00	32.75	68.75	12.00	16.25
M4c								
*E. faecalis*	26.75	+ 100	8.00	33.25	18.75	61.00	23.00	43.75
M15b								
*E. faecalis*	19.25	77.00	22.25	59.00	23.50	27.75	7.50	10.75
M15d								
*E. faecalis*	15.25	86.50	22.25	77.75	25.00	73.00	11.25	17.50
M21a								
*E. faecalis*	12.50	59.75	14.00	39.00	25.00	47.00	8.25	17.50
M21c								
*E. faecalis*	16.00	46.25	11.75	37.50	33.25	58.25	14.25	17.50
M23a								
*E. faecalis*	12.50	64.50	10.75	26.50	29.75	64.00	14.50	18.50
M23c								
*E. faecalis*	12.50	54.25	16.75	41.75	27.00	58.25	18.75	18.75
M25a								
*E. faecalis*	12.50	91.25	11.75	36.75	23.75	56.25	14.50	33.25
M25c								
*E. faecalis*	12.50	72.25	11.50	37.50	18.75	90.25	7.50	23.75
M28a								
*E. faecium*	10.50	48.50	NA	NA	25.00	64.50	NA	NA
M28d								
*E. faecium*	8.50	37.50	NA	NA	26.25	78.75	NA	NA
M29b								
*E. faecalis*	12.50	41.00	10.50	72.25	23.50	62.50	5.75	6.50
M29c								
*E. faecium*	12.50	39.25	NA	NA	18.75	54.75	NA	NA
M32a								
*E. faecalis*	17.50	79.25	16.75	41.75	27.00	66.75	5.75	12.50
M32b								
*E. faecalis*	16.25	69.25	15.00	40.75	31.25	60.50	7.00	7.00
*E. faecalis* ATCC® 29,212	15.00	+ 100	20.00	60.25	25.00	29.25	16.75	68.75
Average	14.90	66.63	13.60	42.34	24.61	63.09	12.46	21.87
SD	4.10	19.55	4.31	16.61	4.64	13.22	5.16	11.33

*M* mouth, *SD* standard deviation, *NA* not applicable, *MIC* minimum inhibitory concentration, *MBC* minimum bactericidal concentration, *MBIC* minimum biofilm inhibitory concentration, *MBEC* minimum biofilm eradication concentration

**Table 2:** Results of statistical analysis with mixed models, comparing MIC, MBC, MBIC and MBEC values of nisin and supplemented guar-gum gel solutions

	MIC	MBC	MBIC	MBEC
Chisq	162.16	7.64	0.32	18.30
*p*-value	< 0.05^*^	0.01^*^	0.57	< 0.05^*^

*Chisq* chi square statistic value, *MIC* minimum inhibitory concentration, *MBC* minimum bactericidal concentration, *MBIC* minimum biofilm inhibitory concentration, *MBEC* minimum biofilm eradication concentration

^*^statistical difference

Regarding the nisin solution, MBC values were almost 5-fold higher than the MIC ones. The average MBC value was 66.63 ± 19.55 μg/mL, with one isolate presenting a MBC value higher than 100 μg/mL. When incorporated in the guar-gum gel, nisin MBC values were also significantly different (*p*-value =0.01), with the mean MBC value being 63.09 ± 13.22 μg/mL, as shown in Tables 1 and 2.

### Minimum biofilm inhibition and eradication concentration of nisin and the supplemented guar-gum gel

MBIC and MBEC values were determined for the biofilm producing strains (*n* = 17), since M28a, M28d and M29c isolates were previously found not to be biofilm-producers [9].

All isolates tested were considered to be susceptible to both nisin and the supplemented guar-gum gel solutions. MBIC and MBEC values for each strain are presented in Table 1. For the nisin solution, MBIC values ranged from 8.00 to 22.25 μg/mL, with an average value of 13.60 ± 4.31 μg/mL. For the supplemented guar-gum gel solution MBIC values ranged from 5.75 to 23.00 (μg/mL), with an average MBIC value of 12.46 ± 5.16 (μg/mL). No significant differences were observed between MBIC values of both solutions (Table 2).

Regarding MBEC determination, values obtained were 3 and 2-fold higher than the MBIC ones obtained for the nisin and the supplemented guar-gum gel solutions, respectively (Table 1). For the nisin solution, values ranged from 13.00 to 77.75 μg/mL, with an average value of 42.34 ± 16.61 μg/mL, while for the supplemented guar-gum gel solution, MBEC values ranged from 6.50 to 68.75 μg/mL, with an average value of 21.87 ± 11.33 μg/mL. Statistical differences were found between MBEC values for both solutions (*p*-value< 0.05).

### Antimicrobial potential of the nisin-supplemented veterinary toothpaste

**Fig. 1:**
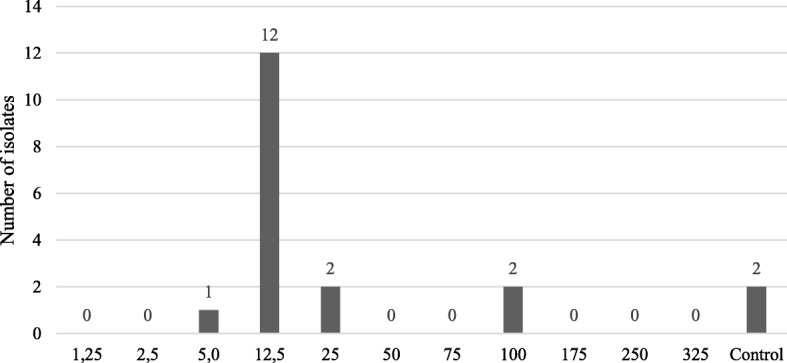
Inhibitory activity of supplemented toothpaste in the enterococci collection. Distribution of the number of susceptible isolates by nisin concentration in the supplemented toothpaste. The non-supplemented toothpaste was used as control

**Fig. 2:**
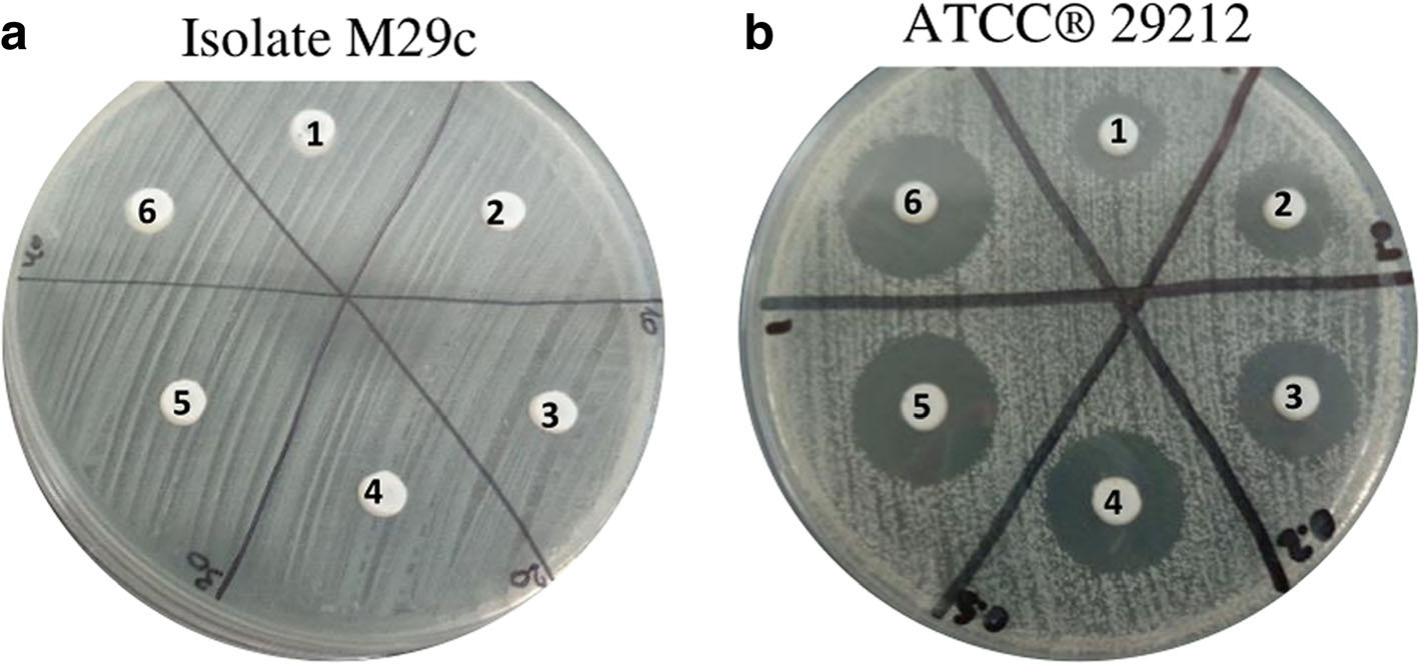
Antimicrobial activity of a supplemented veterinary toothpaste determined by an agar-well diffusion assay. **a** A non-susceptible isolate (*E. faecalis* M29c). **b** A susceptible isolate (*E. faecalis* ATCC® 29,212), increasing nisin concentrations in which produced increasing inhibition zone diameters (1-non-supplemented toothpaste; 2- supplemented toothpaste at 1.25 μg/mL; 3- supplemented toothpaste at 2.5 μg/mL; 4- supplemented toothpaste at 5 μg/mL; 5- supplemented toothpaste at 12.5 μg/mL; 6- supplemented toothpaste at 25 μg/mL)

When supplemented with nisin, the toothpaste presented an inhibitory effect against 95% (19/20) of the isolates under study. Considering MIC as the lowest concentration of nisin incorporated in the toothpaste to produce an inhibition halo, 5% of the isolates presented a MIC value of 5 μg/mL, 60% of 12.5 μg/mL, 10% of 25 μg/mL and 10% of 100 μg/mL (Fig. 1). As expected, increasing nisin concentrations produced higher inhibition zone diameters, which ranged from 11 to 20.33 mm (Fig. 2b).

The supplemented toothpaste was not able to produce an inhibitory effect regarding one isolate (5%) (Fig. 2a).

The non-supplemented toothpaste showed inhibitory action against 10% of the isolates (2/20) (Figs. 1 and 2b).

## Discussion

PD is an inflammatory disease with high prevalence in dogs. Its physiopathology, associated with an inefficient prophylaxis, contribute to the high prevalence and severity of this disease [2]. Among the bacteria implicated with the formation of dental biofilms that are responsible for PD onset, *Enterococcus* spp. was recently classified by WHO as high priority pathogen for the development of new antimicrobial drugs. As this bacterium frequently presents a multi-drug resistant (MDR) profile and have the potential to promote severe systemic complications [8, 9], alternative strategies to conventional antimicrobials are urgent for PD control in dogs [12, 13]. Nisin is an AMP active against a wide range of Gram-positive bacteria and some Gram-negative bacteria [13, 19, 20]. This AMP also presents immunomodulatory effects, wound healing capacity, and antibiofilm properties, being a promising candidate for PD control in dogs [28].

In this study, the ability of nisin to inhibit and eradicate enterococci isolated from the oral cavity of dogs with PD was assessed. Results showed that all bacterial isolates, including planktonic and biofilm producing strains, were susceptible to the nisin solution, in agreement with previous studies [18, 29, 30]. As expected, MBC and MBEC values were higher than MIC and MBIC values, respectively. However, nisin exhibited antibiofilm activity (MBIC and MBEC) at concentrations that were lower than the ones required for planktonic cells inhibition, revealing a possible “nonclassical” mechanism of action targeting the biofilm mode of growth, as described by Batoni and collaborators (2016) [28].

Despite the excellent in vitro antimicrobial activity of nisin, its delivery remains a challenge, as previously described [20, 24]. Therefore, two delivery systems with the potential for topical administration of nisin to the oral cavity of dogs were tested: a guar-gum gel and a veterinary toothpaste.

Guar-gum is a non-ionic natural polysaccharide widely explored in biomedical and industrial research [25]. This safe, stable, biodegradable and biocompatible compound is being evaluated as a potential drug-controlled release compound [19, 25]. In this study, nisin was incorporated in a 1.5% (*w*/*v*) guar-gum gel formulation [20] and the inhibitory activity of this formulation was tested against the enterococci collection, revealing a positive antimicrobial activity against both planktonic and biofilm cells. In fact, when compared with the MIC for the nisin solution, the values were statistically different (*p*-value< 0.05), with the nisin-supplemented guar-gum gel solution presenting a higher mean value (Tables 1 and 2).

Regarding MBC, the values obtained for the supplemented guar-gum gel were two-fold higher than MIC ones. However, they were lower than the MBC values for the nisin solution, being this difference statistically significant (p-value = 0.01). According to Levison and collaborators (2009), an antimicrobial agent is classified as bactericidal if its MBC is no more than four times the MIC [31]. In this study, nisin solution presented a bactericidal action against 42.86% of the enterococci tested, while when incorporated in guar-gum gel, it had a bactericidal effect against 95% of the isolates. These results suggest a protective role of guar-gum regarding nisin, leading to a higher bactericidal effect. This can be explained by the physiochemical properties of guar gum, which remains stable at acidic pH values, in which nisin has optimal activity [26]. Knowing if a compound has a bactericidal or a bacteriostatic activity is essential for a precise antimicrobial therapy [32]. In fact, some cases of PD may require antimicrobial therapy, mainly in moderate to severe cases and when there are other concomitant diseases [4]. Being a polymicrobial disease, PD may benefit from a combined antimicrobial therapy [2, 3, 32]. Usually, the antimicrobials more used in PD are amoxicillin/clavulanate, ampicillin, spiramycin, metronidazole, clindamycin and doxycycline, among others [4, 5]. Apart from clindamycin and doxycycline that are bacteriostatic, the others have a bactericidal action. Reports suggest that, a synergistic effect is expected with the combination of two bactericidal drugs, but, on the other hand, bacteriostatic antimicrobials frequently antagonize the action of bactericidal compounds [32]. As we describe, supplemented guar-gum gel as a bactericidal effect against 95% of the isolates showing potential to be combined efficiently with other bactericidal compounds, as most of the antimicrobials used in PD treatment.

Considering biofilm cells, nisin antibiofilm activity was maintained and even increased when incorporated in the guar-gum gel solution. As observed in Table 1, MBIC and MBEC values of the supplemented guar-gum gel solution were lower than the ones of the nisin solution. In fact, biofilm eradication concentration (MBEC) values of the supplemented guar-gum gel solution were statistically different (*p*-value < 0.05) from the ones of the nisin solution, confirming the capacity of the guar-gum gel in improving the bactericidal activity of nisin towards biofilm cells, as observed for their planktonic counterparts. Besides contributing for an increase in nisin’s bactericidal activity, the proven bio-adhesiveness capacity and low price of the guar-gum gel makes this potential AMP delivery system an optimal candidate for further studies in several biomedicine areas, including odontology.

Another nisin delivery system evaluated in this study was a commercially available veterinary toothpaste (C.E.T.® Enzymatic Toothpaste for Dogs and Cats, Virbac), as toothbrushing represents an important home oral hygiene method for PD prevention, promoting the mechanical reduction of the dental plaque [5, 10, 27]. This toothpaste has a complex composition, with a dual-enzymatic action based on the activity of two proteins, glucose oxidase and lactoperoxidase. Glucose oxidase oxidizes glucose to gluconolactone and hydrogen peroxide, that activates the lactoperoxidase system which oxidises thiocyanate to hypothiocyanite, which is also an antibacterial agent [33]. In our study, the non-supplemented toothpaste showed antimicrobial activity against 10% of the isolates (2/20) (Figs. 1 and 2b); however, this antimicrobial activity has increased to 60% (12/20) when supplemented with nisin at 12.5 μg/mL, and to 95% (19/20) when supplemented with nisin at 100 μg/mL.

Only one isolate was not susceptible to the supplemented toothpaste (Fig. 2 a). Regarding the genetic characteristics of this isolate, apart from being non-biofilm producer, no major differences were detected between the remaining isolates [9]. In fact, this isolate (M29c) was both susceptible to nisin and supplemented guar-gum gel solutions, with MIC mean values of 12.50 μg/mL and 18.75 μg/mL, respectively. So, its resistance capacity regarding the supplemented toothpaste may be related with a specific unknown property or may be due to different levels of expression of the *efaA*_*fm*_ and *ace* genes (Table 3), which may contribute for its protection against the antimicrobial activity of the supplemented toothpaste.

**Table 3:** Characterization of the oral enterococci collection regarding antimicrobial resistance and virulence profiles [9]

Strain Identification	Virulence genes	Resistance phenotype
M2b		
*E. faecalis*	*efaA*_*fs*_ *-gelE-gls24-ebpA-ebpB-ebpC*	CTX-TE-DA-QD
M2c		
*E. faecalis*	*efaA*_*fs*_ *-gelE-gls24-ebpA-ebpB-ebpC*	CTX-TE-DA-QD
M3b		
*E. faecalis*	*efaA*_*fs*_ *-esp-agg-ace-gls24-ebpA-ebpB ebpC*	CTX-TE-CN-DA-CIP-LEV-QD-S
M3d		
*E. faecalis*	*efaA*_*fs*_ *-esp-agg-ace-gls24-ebpA-ebpB-ebpC*	CTX-TE-CN-DA-CIP-LEV-QD
M4a		
*E. faecalis*	*efaA*_*fs*_ *-gelE-ebpA-ebpB-ebpC*	CTX-DA-QD
M4c		
*E. faecalis*	*efaA*_*fs*_ *-gelE-ace-gls24-ebpA-ebpB-ebpC*	CTX-TE-DA-C-QD
M15b		
*E. faecalis*	*efaA*_*fm*_ *-agg-ace-gls24-ebpA-ebpB-ebpC*	CTX-TE-DA-E-QD
M15d		
*E. faecalis*	*efaA*_*fm*_ *-agg-ace-gls24-ebpA-ebpB-ebpC*	CTX-TE-CN-DA-E-QD-S
M21a		
*E. faecalis*	*efaA*_*fs*_ *-cyIA-gls24-ebpA-ebpB-ebpC,*	CTX-TE-DA-QD
M21c		
*E. faecalis*	*efaA*_*fs*_ *-cyIA-gls24-ebpA-ebpB-ebpC,*	CTX-TE-CN-DA-QD
M23a		
*E. faecalis*	*efaA*_*fs*_ *-agg-cyIA-ace-gls24-ebpA-ebpB-ebpC*	CTX-TE-CN-DA-C-E-QD-S
M23c		
*E. faecalis*	*efaA*_*fs*_ *-agg-cyIA-ace-gls24-ebpA-ebpB-ebpC*	CTX-TE-CN-DA-C-E-QD-S
M25a		
*E. faecalis*	*efaA*_*fs*_ *-gls24-ebpA-ebpB-ebpC*	CTX-TE-CN-DA-QD-S
M25c		
*E. faecalis*	*efaA*_*fs*_ *-gls24-ebpA-ebpB-ebpC*	CTX-TE-CN-DA-QD-S
M28a		
*E. faecium*	*efaAfm-acm*	CTX-TE-DA
M28d		
*E. faecium*	*efaAfm-acm*	CTX-TE-DA
M29b		
*E. faecalis*	*efaA*_*fs*_ *-cyIA-ace-gelE-gls24-ebpA-ebpB-ebpC*	CTX-TE-CN-DA-QD-S
M29c		
*E. faecium*	*efaAfm-acm*	CTX-TE-DA
M32a		
*E. faecalis*	*efaA*_*fs*_ *-gelE-ace-ebpA-ebpB-ebpC*	CTX-TE-CN-DA-QD
M32b		
*E. faecalis*	*efaA*_*fs*_ *-gelE-ace-ebpA-ebpB-ebpC*	CTX-TE-CN-DA-QD

*M* mouth, Virulence determinants: *ace* adhesin of collagen from *E. faecalis*, *acm* adhesin of collagen from *E. faecium*, *agg*-aggregation substance, *cylA* cytolysin activa-tor, *ebpABC* pili-like from *E. faecalis*, *efaA*_*fs*_ cell wall adhesion from *E. faecalis*, *efaA*_*fm*_ cell wall adhesion from *E. faecium*, *esp*-cell wall-associated protein, *gelE* gelatinase, *CTX* cefotaxime, *TE* tetracycline, *CN* gentamycin, *QD* quinupristin/dalfopristin, *DA* Clindamycin, *CIP* Ciprofloxacin, *LEV* Levofloxacin, *C* Chloramphenicol, *S* Streptomycin

As expected, the incorporation of increasing nisin concentrations in the toothpaste promoted increasing inhibition zone diameters (Fig. 2b), which ranged from 11 to 20.33 mm. Therefore, the toothpaste has also the potential to be used as a delivery system for nisin topical administration to the oral cavity of dogs, as it showed the capacity to maintain the antimicrobial activity of this AMP and allowed its dispersion, as demonstrated by the well-diffusion assay.

## Conclusion

PD has a significant health impact in dogs. The multifactorial ethology associated to a complex pathogenesis hinders the medical approach to this disease. In fact, biofilms are microbial communities that display unique characteristics compared with their planktonic counterparts, which must be accurately considered when evaluating the potential of biofilm prevention or control strategies. Given the intrinsic resistance of biofilms to antimicrobial therapy, the development of new compounds or novel antimicrobial protocols, able to target not only planktonic cells, but also specific features of this sessile lifestyle are urgent [14, 15, 30]. Described by several authors as antibiofilm compounds, AMP, such as nisin, are promising agents for PD control in dogs [22, 23]. A great deal of effort is being carried out to overcome the problems associated with AMP use in therapeutics such as the development of efficient delivery systems, being expected that they will become the drug of choice for emerging bacterial infections in the future [12, 14, 17, 28].

Nisin seems to be an appropriate AMP candidate for dental plaque control in dogs, as it not only presented effective antimicrobial activity against all the enterococci tested, but also it kept its inhibitory activity when incorporated in the two delivery systems tested. Despite a more consistent and effective inhibitory activity has been observed with supplemented guar-gum gel, supplemented toothpaste remains a very interesting product to be considered in PD management. Supplemented toothpaste may be combined with the supplemented guar-gum gel, the first for home dental care and the second for therapeutic purposes. Therefore, this innovative therapeutic strategy may in the future substitute or complement antibiotherapy, aiming at reducing antibiotics’ administration for bacterial control in the veterinary setting.

## Methods

### Bacterial collection

A collection of 20 enterococci, including planktonic and biofilm-producer strains, obtained in a previous study from the oral cavity of dogs diagnosed with PD [9], were used as bacterial models, to evaluate the antimicrobial activity of nisin and of nisin incorporated in two delivery systems.

All isolates were previously characterized regarding clonality, antimicrobial resistance and virulence profiles, including biofilm-forming ability (Table 3) [9]. One human reference strain, *Enterococcus faecalis* ATCC® 29212, was also included in this study as a control strain.

### Nisin solutions

A nisin stock solution (1000 μg/mL, 40,000 IU/mL) was obtained by dissolving 1 g of nisin powder (2.5% purity, 1000 IU/mg, Sigma-Aldrich, USA) in 25 mL of HCl (0.02 M) (Merck, Germany) [20]. This stock solution was filtered using a 0.22 μm Millipore filter (Frilabo, Portugal) and serial dilutions were prepared in distilled sterile water, with the following concentrations: 750, 625, 500, 375, 250, 125, 50, 25 and 12.5 μg/mL. Working solutions were kept at 4 °C during the study.

### Supplemented guar-gum gel solutions

A 1.5% guar-gum gel (*w*/*v*) solution was prepared by dissolving 0.75 g of guar-gum (Sigma-Aldrich, USA) in 50 mL of sterile distilled water, and heat sterilized by autoclave [20]. Nisin dilutions were incorporated within the guar-gum gel in a proportion of 1:1, obtaining a 0.75% gel (w/v).

### Supplemented toothpaste solutions

The veterinary toothpaste used in this study was C.E.T.® Enzymatic Toothpaste for Dogs and Cats (Virbac®). Solutions of toothpaste supplemented with nisin were prepared in a 2:1 proportion. Working solutions were stored at 4 °C and used only for 7 days. Non-supplemented toothpaste diluted in distilled sterile water (2:1) was used as negative control.

### Minimum inhibitory concentration (MIC) and minimum bactericidal concentration (MBC) determinations

MIC and MBC values for nisin and the supplemented guar-gum gel solutions were determined using a broth microdilution technique adapted from the Clinical and Laboratory Standards Institute (CLSI) guidelines [34].

Strains were grown in a nonselective Brain Heart Infusion (BHI) agar medium (VWR Chemicals, Belgium) at 37 °C for 24 h. Bacterial suspensions with ≈ 10^8^ CFU/mL were prepared directly from fresh agar-plate cultures using a 0.5 McFarland standard in sterile normal saline, after which were diluted in Tryptic Soy Broth (TSB) (VWR Chemicals, Belgium) to a concentration of ≈10^6^ CFU/mL.

For the determination of MIC and MBC values, all the wells of a 96-well flat-bottomed polystyrene microtiter plate (VWR® Tissue culture plates), except for the negative control (with broth only), were inoculated with 180 μL of the enterococcal suspensions and 20 μL of the nisin solutions, or with 160 μL of bacterial suspension and 40 μL of the guar-gum gel solutions. In both cases, the final concentrations of nisin in the wells ranged from 1.25 to 100 μg/mL per well. A positive control containing only bacterial suspension was also included. Microplates were statically incubated for 24 h at 37 °C and MIC was determined as the lowest concentration of nisin that visually inhibited microbial growth [20].

MBC value was determined by inoculating 3 μL of the bacterial suspensions of the wells where no growth was observed on Tryptic Soy Agar (TSA) (VWR Chemicals, Belgium) plates and incubated at 37 °C for 24 h. MBC was determined as the lowest nisin concentration at which no colonies were observed after incubation [20].

All assays were performed in triplicate, in independent days and including 10% of replicates to assure results representability.

### Minimum biofilm inhibitory concentration (MBIC) and minimum biofilm eradication concentration (MBEC) determinations

A modified version of the Calgary Biofilm Pin Lid Device was used to evaluate the susceptibility to nisin of bacteria embedded in a 48 h biofilm [35]. MBIC and MBEC determinations were performed as described by Tremblay et al. (2014) with some modifications [36]. Briefly, 200 μL of 10^6^ CFU/mL bacterial suspensions in TSB supplemented with 0.25% glucose (*w*/*v*) (Merck, USA) were deposited in a 96-well microplate (Nunc™, Thermo Scientific), covered with a peg lid (Nunc™ Immuno TSP Lids, Thermo Scientific™) and statically incubated for 48 h at 37 °C. After incubation, pegs were washed three times with sterile distilled water and transferred to 96-well plates containing 180 μL of TSB supplemented with glucose and 20 μL of the nisin solutions, or 160 μl of TSB with glucose and 40 μL of the supplemented guar-gum gel solutions. Then, the plate was incubated for 24 h at 37 °C after which the MBIC value was determined as the lowest concentration of nisin to inhibit bacterial growth, as detected by direct observation [20].

Pegs were then washed again three times with sterile distilled water and transferred to a 96-well plate containing 200 μL of TSB supplemented with glucose. These plates were sealed and incubated in an ultrasonic bath (Gramt, Ultrasonic Bath, MXB14) for 15 min at high frequency (50–60 Hz). Afterwards, the peg lid was substituted by a conventional one, and the plate was incubated at 37 °C for 24 h, after which optical density at 600 nm (OD600) was measured using a microtiter plate reader (BMG Labtech, FLUOstar OPTIMA) [20]. MBEC value was considered as the lowest concentration of nisin to promote bacterial suspensions with an OD600 <  0.1 [36].

Experiments were conducted in triplicate, in independent days. Additionally, 10% of replicates were also tested to assure results representability.

### Evaluation of the antimicrobial potential of the nisin-supplemented toothpaste

An agar-well diffusion assay was used to evaluate the inhibitory ability of toothpaste solutions supplemented with different concentrations of nisin, selected based on the MIC and MBC values [37].

Nisin was incorporated in the toothpaste in a 1:2 proportion, aiming to obtain solutions with final nisin concentrations of 325, 250, 175, 100, 75, 62.5, 50, 37.5, 25, 12.5, 5, 2.5 and 1.25 μg/mL. Non-supplemented toothpaste diluted with distilled sterile water was used as negative control.

A 10^8^ CFU/mL bacterial suspension was prepared for each isolate and evenly spread onto the surface of TSA plates. Then, six wells per plate were performed, with a standardized volume. After, 40 μL of each toothpaste solution were placed in the wells and the plates were incubated for 24 h at 37 °C. After incubation, plates were observed for the detection of inhibition halos around each well, which diameters were measured.

All assays were conducted in triplicate, in independent days. Additionally, 10% of replicates were performed to assure results representability.

### Statistical analysis

Data statistical analysis was carried out using RStudio® software version 1.1.383 (Boston, USA) and Microsoft Excel 2016®. Linear mixed models were used for statistical analysis of the MIC, MBC, MBIC and MBEC values obtained for nisin and supplemented guar-gum gel solutions.

Quantitative variables are expressed as mean values ± standard deviation. A confidence interval of 95% was considered, with a *p*-value ≤0.05 indicating statistical significance.

## Abbreviations

AMP: Antimicrobial peptide
ATCC: American type culture collection
BHI: Brain heart infusion
CLSI: Clinical and laboratory standards institute
EFSA: European food safety authority
FAO: Food and agriculture organization
FDA: Food and drug administration
MBC: Minimum bactericidal concentration
MBEC: Minimum biofilm eradication concentration
MBIC: Minimum biofilm inhibitory concentration
MDR: Multi-drug resistant
MIC: Minimum inhibitory concentration
OD600: Optical density at 600 nm
PD: Periodontal disease
TSA: Tryptic soy agar
TSB: Tryptic soy broth
WHO: World health organization
